# Repurposing of the analgesic Neurotropin for MASLD/MASH treatment

**DOI:** 10.1097/HC9.0000000000000480

**Published:** 2024-07-18

**Authors:** Takashi Tsuchiya, So Yeon Kim, Michitaka Matsuda, Jieun Kim, Alexsandr Stotland, Mitsuru Naiki, Ekihiro Seki

**Affiliations:** 1 Department of Medicine, Cedars-Sinai Medical Center, Los Angeles, California, USA; 2 Department of Pharmacological Research, Institute of Bio-Active Science, Nippon Zoki Pharmaceutical Company Ltd., Osaka, Japan; 3 Department of Biomedical Sciences, Cedars-Sinai Medical Center, Los Angeles, California, USA

## Abstract

**Background:**

The prevalence of metabolic dysfunction–associated steatotic liver disease (MASLD) has increased in recent decades. Approximately 25% of patients with MASLD progress to metabolic dysfunction–associated steatohepatitis, which is characterized by hepatic steatosis plus hepatocyte damage, inflammation, and fibrosis. We previously reported that Neurotropin (NTP), a drug used for relieving pain in Japan and China, inhibits lipid accumulation in hepatocytes by preventing mitochondrial dysfunction. We hypothesized that inhibiting hepatic steatosis and inflammation by NTP can be an effective strategy for treating MASLD and tested this hypothesis in a MASLD mouse model.

**Methods:**

Six-week-old C57BL/6NJ male mice were fed a normal diet and normal drinking water or a high-fat diet with high fructose/glucose water for 12 weeks. During the last 6 weeks, the mice were also given high-dose NTP, low-dose NTP, or control treatment. Histologic, biochemical, and functional tests were conducted. MitoPlex, a new proteomic platform, was used to measure mitochondrial proteins, as mitochondrial dysfunction was previously reported to be associated with MASLD progression.

**Results:**

NTP inhibited the development of hepatic steatosis, injury, inflammation, and fibrosis induced by feeding a high-fat diet plus high fructose/glucose in drinking water. NTP also inhibited HSC activation. MitoPlex analysis revealed that NTP upregulated the expression of mitochondrial proteins related to oxidative phosphorylation, the tricarboxylic acid cycle, mitochondrial dynamics, and fatty acid transport.

**Conclusions:**

Our results indicate that NTP prevents the development of hepatic steatosis, injury, and inflammation by preserving mitochondrial function in the liver and inhibits liver fibrosis by suppressing HSC activation. Thus, repurposing NTP may be a beneficial option for treating MASLD/metabolic dysfunction–associated steatohepatitis.

## INTRODUCTION

The prevalence of metabolic dysfunction–associated steatotic liver disease (MASLD) has increased dramatically in recent decades. MASLD has become one of the most common liver disorders, affecting ~25% of adults in Western and Asian countries.^1^^–^^3^ MASLD is often associated with other metabolic disorders, including obesity, diabetes, hyperlipidemia, and cardiovascular disease. Approximately 20%–25% of patients with MASLD eventually develop metabolic dysfunction–associated steatohepatitis (MASH),^1^^–^^3^ characterized by steatosis with hepatocyte ballooning, liver inflammation, and fibrosis. MASH may progress to fibrosis and, ultimately, cirrhosis. Fibrosis is a crucial factor determining the prognosis of patients with MASH. Importantly, MASLD, even without cirrhosis, increases the risk of HCC.^4^

Recently, resmetirom, an agonist of thyroid hormone receptor-β, has been approved by the US Food and Drug Administration (FDA) for the treatment of MASLD.^5^ Various new drugs are still under investigation for treating MASLD.^6^ Repurposing existing FDA-approved drugs is also an option for developing drugs for treating MASLD because this approach can save considerable time and costs when developing effective therapies. In this study, we evaluated the potential of repurposing Neurotropin (NTP) for the treatment of MASLD.

NTP is a nonprotein fraction extracted from inflamed rabbit skin following the administration of the vaccinia virus. NTP has been widely used in Japan and China for more than 50 years as an analgesic for chronic skeletal muscle and neuropathic pain.^7^^–^^9^ NTP contains various bioactive molecules, including nucleic acids, amino acids, and sugars.^10^ Although the biologically active components responsible for its beneficial effects have not been identified, NTP exerts analgesic effects through the activation of the descending pain inhibitory system and induction of brain-derived neurotrophic factor.^11^^,^^12^ Also, NTP has cytoprotective activity against TNFα- and IL-1β–induced hepatocyte apoptosis, as well as cancer chemotherapy–mediated neurodegeneration and neuropathy.^13^^,^^14^ We and others previously showed that NTP inhibits NF-κB, Jun kinase (JNK), extracellular signal-regulated kinase (ERK), and p38 mitogen-activated protein kinase signaling pathways and the induction of TNFα, IL-6, and cyclooxygenase (COX)-2 in neurons, microglia, and hepatocytes.^14^^–^^16^ Moreover, we previously reported that NTP inhibits lipid accumulation and the production of mitochondrial reactive oxygen species (ROS) and increases the mitochondrial membrane potential in mouse primary hepatocytes challenged with free fatty acid (FFA) in vitro.^17^^–^^19^ We also reported that NTP promotes mitochondrial biogenesis and turnover by upregulating peroxisome proliferator-activated receptor gamma coactivator 1-beta (PGC-1β) and AMPK activation.^17^ These anti-inflammatory and cytoprotective effects and the beneficial effect on mitochondrial functions suggest that NTP may be useful for treating MASLD. Therefore, we hypothesized that NTP would have beneficial therapeutic effects on fatty liver disease in vivo.

In this study, we used a mouse animal model to investigate the potential effectiveness of NTP for treating MASLD/MASH. Our results revealed that NTP reduces hepatocyte lipid accumulation and injury, inflammatory cell infiltration, and HSC activation. Furthermore, NTP has the potential to recover mitochondrial function. Our data suggest a possible repurposed role for NTP in the clinical management of patients with MASLD/MASH.

## METHODS

### Reagents

NTP was provided by Nippon Zoki Pharmaceutical Company Ltd. Obeticholic acid (OCA) was purchased from MedChemExpress LLC. Kits for measuring alanine transaminase and aspartate aminotransferase were obtained from Thermo Scientific. Kits for serum and hepatic triglyceride, cholesterol, and FFAs were purchased from Pointe Scientific Inc., FUJIFILM Medical Systems USA Inc., and Abcam, respectively.

### Mice

Six-week-old C57BL/6NJ male mice were purchased from Jackson Laboratories. Mice received a normal chow diet (ND) (PicoLab Rodent Diet 20 5053; LabDiet) with normal drinking water (NW) or a high-fat diet (HFD; 60% of calories from fat; Cat# S3282, Bio-Serv) with high fructose/glucose drinking water (HFGW) for 12 weeks.^20^ HFGW contained 23.1 g d-(-)-fructose and 18.9 g d-(+)-glucose (Sigma-Aldrich) in 1 L water. All mice were housed in specific pathogen-free conditions at Cedars-Sinai Medical Center. All studies were performed in accordance with the National Institutes of Health recommendations outlined in the Guide for the Care and Use of Laboratory Animals. All animal experiment protocols were approved by the Cedars-Sinai Medical Center Institutional Animal Care and Use Committee.

After 6 weeks of ND/NW or HFD/HFGW feeding, each animal received oral administration of NTP or OCA once per day for another 6 weeks. OCA, a farnesoid X-activated receptor agonist, was used as the active treatment control.^21^ The mice were randomly assigned to receive vehicle control, OCA (30 mg/kg/d), low-dose NTP (50 Neurotropin units [NU]/kg/d), or high-dose NTP (100 NU/kg/d).

### Immunofluorescence staining

After deparaffinization of the slides from formalin-fixed paraffin-embedded blocks, we performed immunofluorescence staining for the macrophage marker F4/80. Slides were incubated with anti-F4/80 antibody (1:100 in PBS; BioLegend) at 4°C overnight, followed by Alexa Fluor 568-conjugated secondary antibody for 1 hour at room temperature. F4/80-positive areas were measured in 8 high-power (×200) fields per slide. Images were captured using a BZ-X710 Keyence fluorescence microscope and analyzed using ImageJ software.

### HSC isolation and treatment

Primary HSCs were isolated from wild-type C57BL/6NJ mice (Jackson Laboratory) or transgenic mice expressing green fluorescent protein under the control of the *Col1a1* promoter. We used the in situ collagenase-pronase perfusion method as described.^22^ Cells with >90% viability were used for the experiments and cultured overnight in DMEM containing 10% fetal bovine serum. The following day, the medium was changed to serum-free DMEM, after which the cells were treated with NTP (0.2 or 0.4 NU/mL) for 1 hour, followed by 5 ng/mL TGF-β for an additional 48 hours. Immunofluorescence staining for α-smooth muscle actin (α-SMA; Sigma-Aldrich) was performed on cultured cells. Cells were incubated with anti–α-SMA antibody (1:100 in PBS) at 4°C overnight, followed by Alexa Fluor 568-conjugated secondary antibody for 1 hour at room temperature. Images were captured using a BZ-X710 Keyence fluorescence microscope and analyzed using ImageJ software.

### Statistical analysis

Statistical analyses were performed using GraphPad Prism 10.0.2 software. Data are expressed as mean ± SD. Differences between the 2 groups were compared using a 2-tailed, unpaired Student *t* test. Differences between multiple groups were compared using ANOVA, followed by the Bonferroni post hoc analysis. *p* values <0.05 were considered statistically significant.

### Supplemental methods

Please see the Supplemental Methods, http://links.lww.com/HC9/A942, for additional details regarding the reagents, histologic analysis, biochemical assays, human hepatocyte culture and treatment, RNA extraction and analysis (Supplemental Table S2, http://links.lww.com/HC9/A942,) immunoblotting, MitoPlex protein analysis, and Seahorse bioanalyzer assessments.

## RESULTS

### NTP treatment prevents hepatic steatosis and injury in HFD/HFGW-fed mice

**FIGURE 1:**
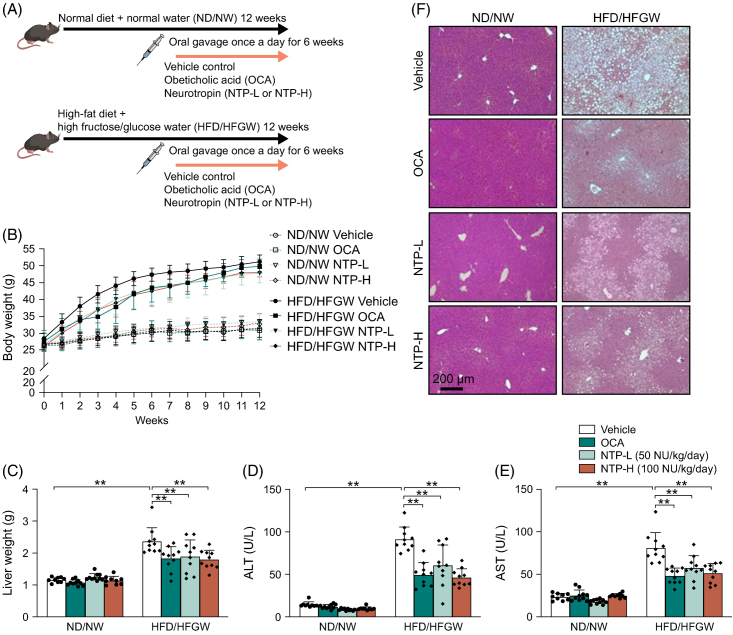
Hepatic steatosis and injury are reduced by NTP treatment in a mouse MASLD/MASH model. (A) Protocol for the mouse MASLD/MASH model. Mice were fed a normal chow diet with normal drinking water (ND/NW) or a high-fat diet with high fructose/glucose drinking water (HFD/HFGW) for a total of 12 weeks. After 6 weeks of diet feeding, the mice also received vehicle control, OCA (30 mg/kg/d), low-dose NTP (NTP-L; 50 NU/kg/d), or high-dose NTP (NTP-H;100 NU/kg/d) for an additional 6 weeks. N = 9–10 in each group. (B) Body weight changes during the 12-week experimental period. (C) Liver weight at week 12. (D, E) Serum ALT and AST levels at week 12. (F) Hematoxylin & eosin staining of liver tissues. Representative pictures are shown. Data are shown as mean ± SD. Significance was determined using 2-way ANOVA, with the Bonferroni post hoc analysis. **p < 0.005. Abbreviations: ALT, alanine aminotransferase; AST, aspartate aminotransferase; MASH, metabolic dysfunction–associated steatohepatitis; MASLD, metabolic dysfunction–associated steatotic liver disease; NTP, Neurotropin; NU, Neurotropin units; OCA, obeticholic acid.

To establish a mouse model of MASLD, C57BL/6NJ wild-type mice were fed an HFD with HFGW for 12 weeks. Mice fed an ND with NW were used as controls. After 6 weeks of ND/NW or HFD/HFGW feeding, the mice continued with the feeding but were also treated with vehicle control, active treatment control (OCA), low-dose NTP (50 NU/kg/d), or high-dose NTP (100 NU/kg/d) for an additional 6 weeks (for a total 12 wk of diet feeding) (Figure 1A). OCA was used as the active treatment control because it has been demonstrated to have beneficial effects in clinical trials of patients with MASH.^23^

Body weight increased more in the HFD/HFGW group than in the ND/NW group, starting at week 2 and continuing throughout the feeding period. The weight in the HFD/HFGW group was ~30% higher than in the ND/NW at week 2 and ~50% higher than in the ND/NW group at week 12 (Figure 1B). In the HFD/HFGW group, there was a trend toward less body weight gain in mice treated with OCA or either dose of NTP, compared to the vehicle control group, but the differences did not reach statistical significance. Liver weight was approximately two-fold higher in HFD/HFGW mice than in ND/NW mice at week 12. In HFD/HFGW mice, liver weight was significantly lower in the OCA and NTP treatment groups compared to the vehicle control group (Figure 1C). Steatosis and elevated serum alanine transaminase and aspartate aminotransferase levels were observed in mice receiving an HFD/HFGW diet (Figures 1D–F). OCA and NTP treatment significantly reduced hepatic steatosis and alanine transaminase and aspartate aminotransferase, compared to the vehicle control, in HFD/HFGW-fed mice (Figures 1D–F). These results, therefore, indicate that NTP is protective against HFD/HFGW-induced hepatic steatosis and injury in mice.

### NTP treatment inhibits hepatic lipid accumulation and lipogenesis in HFD/HFGW-fed mice

**FIGURE 2:**
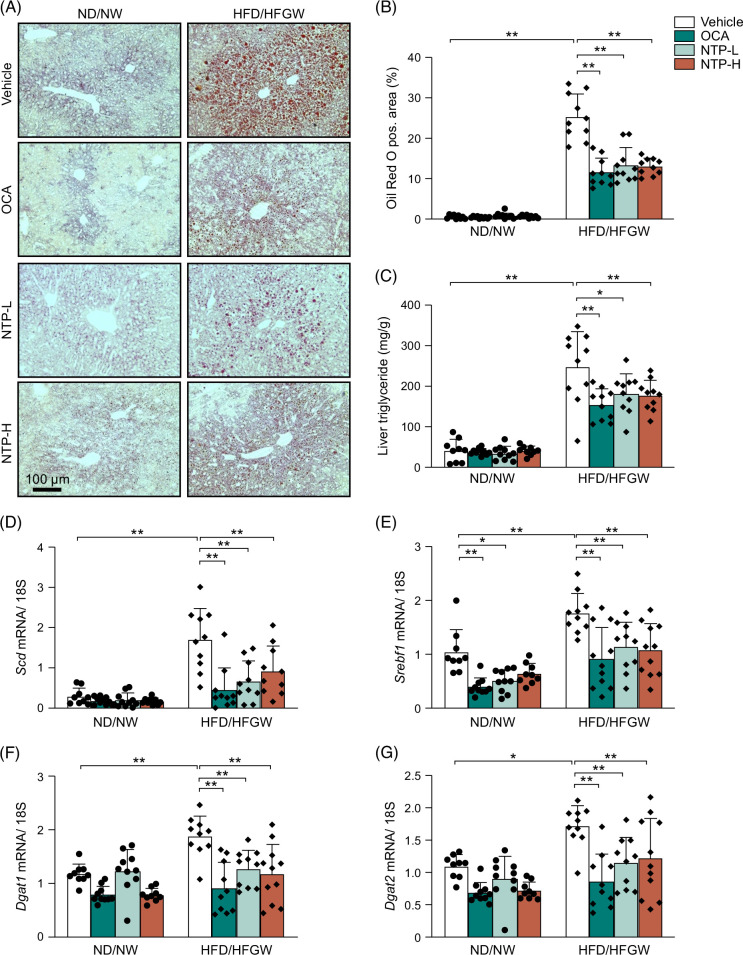
Hepatic lipogenesis is suppressed by NTP treatment in a mouse MASLD/MASH model. Mice were fed a normal chow diet with normal drinking water (ND/NW) or a high-fat diet with high fructose/glucose drinking water (HFD/HFGW) for 12 weeks. Vehicle, OCA, or NTP was administered for the last 6 weeks. N = 9–10 per group. (A) Oil red O staining of liver tissues. Representative pictures are shown. (B) Quantification of Oil Red O–positive areas at week 12. (C) Liver triglyceride levels at week 12. (D–G) Hepatic *Scd*, *Srebf1*, *Dgat1*, and *Dgat2* mRNA expression at week 12 was determined by quantitative real-time PCR. Data are shown as mean ± SD. Significance was determined using 2-way ANOVA, with Bonferroni post hoc analysis. *p < 0.05, **p < 0.005. Abbreviations: MASH, metabolic dysfunction–associated steatohepatitis; MASLD, metabolic dysfunction–associated steatotic liver disease; NTP, Neurotropin; NTP-H, high-dose Neurotropin; NTP-L, low-dose Neurotropin; NU, Neurotropin units; OCA, obeticholic acid; pos., positive.

Next, we analyzed the treatment effect of NTP on HFD/HFGW-induced fat accumulation and lipid metabolism in MASLD. HFD/HFGW-fed mice showed a marked increase in liver fat content, as measured by Oil Red O staining (Figures 2A, B), which was significantly reduced by treatment with NTP or OCA (Figures 2A, B). Consistently, HFD/HFGW feeding led to increased hepatic triglyceride and FFA content, whereas both OCA and NTP led to lower levels of hepatic triglyceride and FFA content in HFD/HFGW-fed mice (Figure 2C, Supplemental Table S1, http://links.lww.com/HC9/A942). Notably, hepatic cholesterol levels increased by HFD/HFGW feeding were further increased with OCA but not with NTP treatment (Supplemental Table S1, http://links.lww.com/HC9/A942). We then examined the expression of genes related to lipogenesis using quantitative real-time PCR. Increased expression of *Scd*, *Srebf1*, *Dgat1*, and *Dgat2* was observed in HFD/HFGW mice and was significantly reduced in mice treated with OCA or NTP (Figures 2D–G). These results suggest that NTP suppresses the expression of lipogenesis genes, thereby reducing hepatic fat content in mice with MASLD.

### NTP treatment suppresses hepatic inflammation in mice induced by HFD/HFGW feeding

**FIGURE 3:**
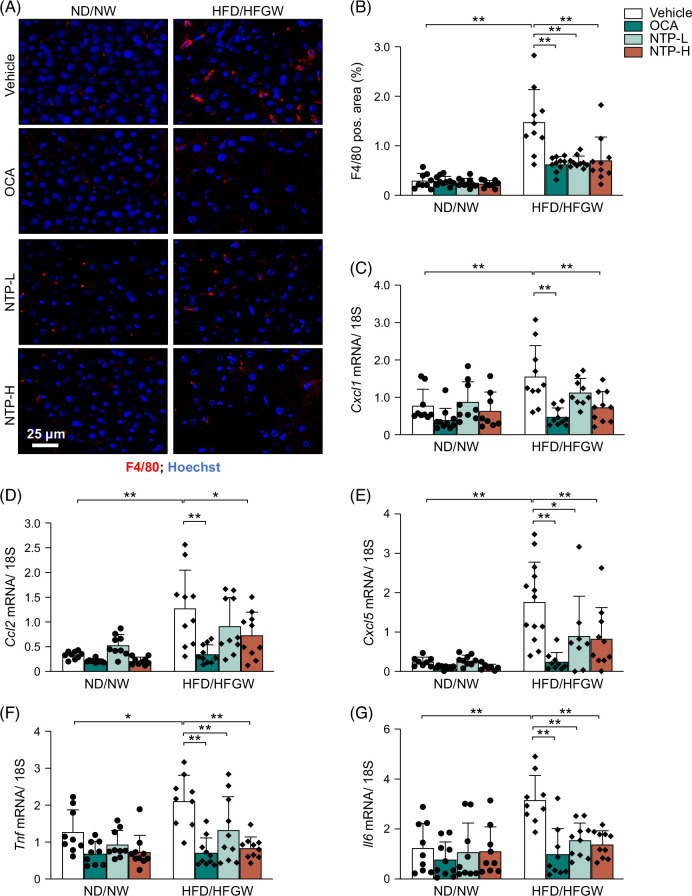
NTP treatment suppresses MASLD/MASH-induced hepatic inflammation in mice. Mice were fed a normal chow diet with normal drinking water (ND/NW) or a high-fat diet with high fructose/glucose drinking water (HFD/HFGW) for 12 weeks. Vehicle, OCA, or NTP was administered for the last 6 weeks. N = 9–10 per group. (A) Immunofluorescence results of liver tissues at week 12, with staining for F4/80 shown in red and for DNA (Hoechst stain) shown in blue. Representative pictures are shown. (B) Quantification of F4/80 staining at week 12. (C–G) Hepatic *Cxcl1*, *Ccl2*, *Cxcl5*, *Tnf*, and *Il6* mRNA expression at week 12 was determined by quantitative real-time PCR. Data are shown as mean ± SD. Significance was determined using 2-way ANOVA, with the Bonferroni post hoc analysis. *p < 0.05, **p < 0.005. Abbreviations: MASH, metabolic dysfunction–associated steatohepatitis; MASLD, metabolic dysfunction–associated steatotic liver disease; NTP, Neurotropin; NTP-H, high-dose Neurotropin; NTP-L, low-dose Neurotropin; NU, Neurotropin units; OCA, obeticholic acid; pos., positive.

**FIGURE 4:**
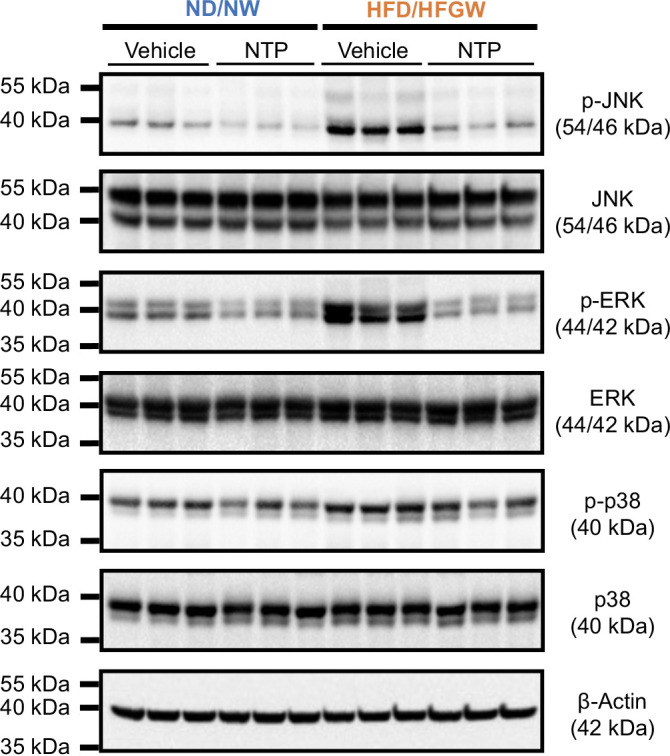
NTP treatment inhibits MASLD/MASH-induced intracellular inflammatory pathways in mice. Protein expression was examined using the lysates from livers of mice fed a normal chow diet with normal drinking water (ND/NW) or a high-fat diet with high fructose/glucose drinking water (HFD/HFGW) for 12 weeks, with treatment of Vehicle control or NTP for the last 6 weeks. Immunoblots for phospho-JNK, total JNK, phospho-ERK, total ERK, phospho-p38, total p38, and β-actin. Three representative protein lysates of each condition were used for immunoblotting. Representative images of 3 independent experiments are shown. Abbreviations: ERK, extracellular signal-regulated kinase; JNK, Jun kinase; MASH, metabolic dysfunction–associated steatohepatitis; MASLD, metabolic dysfunction–associated steatotic liver disease; NTP, Neurotropin.

To investigate the anti-inflammatory effects of NTP in MASLD/MASH mice induced by HFD/HFGW feeding, we examined hepatic macrophage infiltration by immunofluorescence staining for F4/80. Hepatic macrophage infiltration was increased in HFD/HFGW-fed mice, compared to ND/NW mice, and was markedly reduced by treatment with NTP or OCA in HFD/HFGW mice (Figures 3A, B). We next examined chemokine gene expression in the liver. Increased expression of *Cxcl1*, *Ccl2*, *Cxcl5*, *Tnf*, and *Il6* mRNA expression induced by HFD/HFGW feeding was significantly decreased in the OCA and high-dose NTP treatment groups (Figures 3C–G). Then, we examined intracellular inflammatory signaling by assessing the phosphorylation of JNK, ERK, and p38 (Figure 4). HFD/HFGW feeding significantly increased the phosphorylation of JNK, ERK, and p38, whereas the NTP treatment significantly reduced the phosphorylation of JNK, ERK, and p38 (Figure 4). These results indicate that NTP treatment suppresses the expression of inflammatory genes, phosphorylation of JNK, ERK, and p38, and hepatic macrophage infiltration in our MASLD mouse model.

### NTP treatment inhibits the development of liver fibrosis in HFD/HFGW-fed mice

**FIGURE 5:**
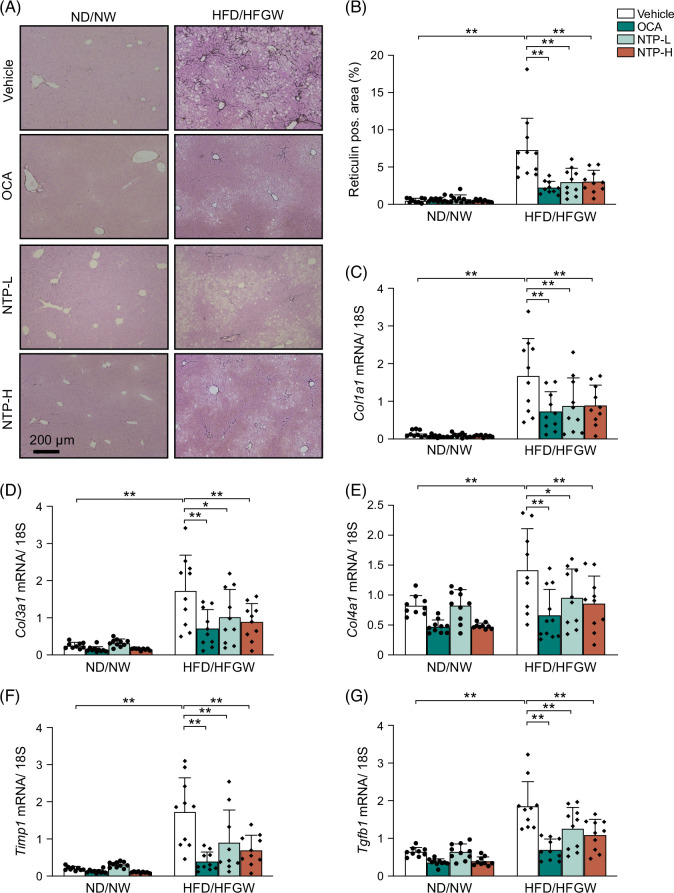
NTP treatment reduces MASLD/MASH-induced hepatic fibrogenesis in mice. Mice were fed a normal chow diet with normal drinking water (ND/NW) or a high-fat diet with high fructose/glucose drinking water (HFD/HFGW) for 12 weeks. Vehicle, OCA, or NTP was administered for the last 6 weeks. N = 9–10 per group. (A) Reticulin staining of liver tissues at 12 weeks. Stained collagen is shown in black. Representative pictures are shown. (B) Quantification of reticulin staining at 12 weeks. (C–G) Hepatic *Col1a1*, *Col3a1*, *Col4a1*, *Timp1*, and *Tgfb1* mRNA expression at 12 weeks was determined by quantitative real-time PCR. Data are shown as mean ± SD. Significance was determined using 2-way ANOVA, with the Bonferroni analysis. *p < 0.05, **p < 0.005. Abbreviations: MASH, metabolic dysfunction–associated steatohepatitis; MASLD, metabolic dysfunction–associated steatotic liver disease; NTP, Neurotropin; NTP-H, high-dose Neurotropin; NTP-L, low-dose Neurotropin; NU, Neurotropin units; OCA, obeticholic acid; pos., positive.

Given fibrosis is the most important determinant of the prognosis of patients with MASH, we evaluated the effects of NTP on fibrosis. Twelve weeks of HFD/HFGW feeding resulted in mild fibrosis, as evaluated by staining for reticulin (which represents type 3 collagen deposition). Reticulin staining showed increased collagen deposition in the HFD/HFGW-fed group, which was drastically reduced by NTP and OCA (Figures 5A, B). To further support these histologic findings, we examined the expression of several fibrogenic genes: *Col1a1*, *Col3a1*, *Col4a1*, and *Timp1*. Consistent with the histology results, fibrogenic gene mRNA expression was upregulated by HFD/HFGW feeding and significantly reduced by NTP or OCA (Figures 5C–F). mRNA expression of *Tgfb1*, which encodes TGF-β, a potent fibrogenic cytokine that induces HSC activation, was also decreased in the OCA and NTP treatment groups (Figure 5G). These data demonstrate that NTP treatment ameliorates liver fibrosis and reduces the expression of fibrogenic genes in a MASLD/MASH mouse model.

### NTP treatment inhibits the activation of mouse primary HSCs induced by TGF-β

**FIGURE 6:**
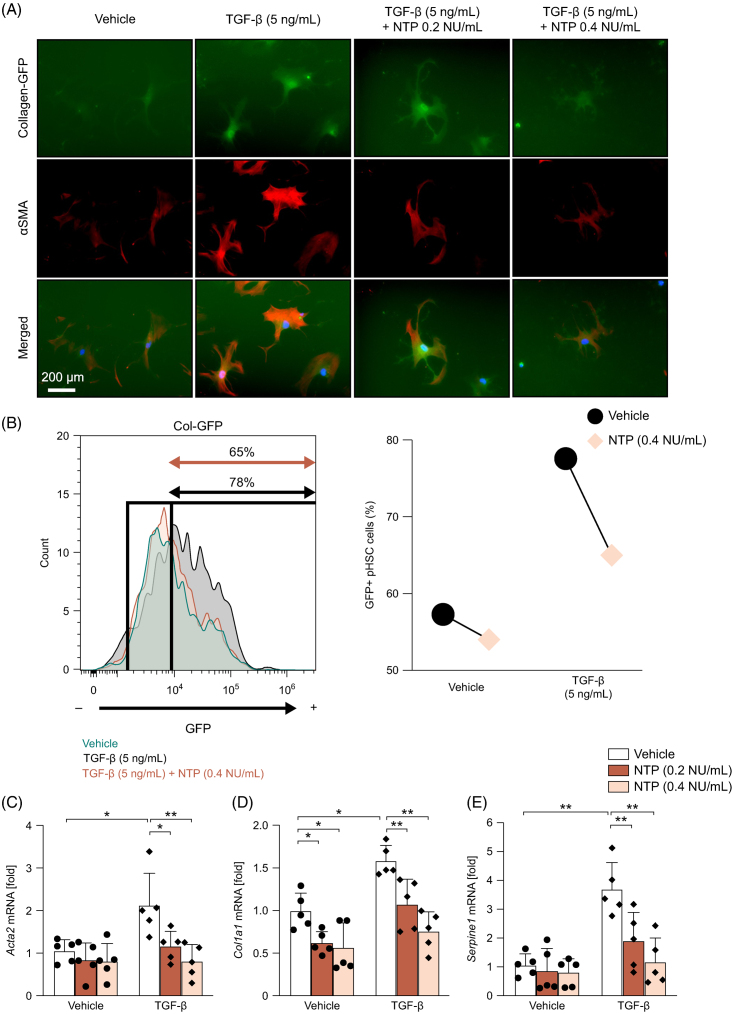
TGF-β–induced fibrogenic response is inhibited by NTP treatment in primary HSCs. Mouse primary HSCs were isolated from *Col1a1-*green fluorescent protein (GFP) reporter transgenic mice. One hour after pretreatment with vehicle or NTP (0.2 or 0.4 NU/mL), HSCs were treated with 5 ng/mL TGF-β for 24 hours. (A) GFP signals and immunofluorescent staining for α-SMA were visualized by fluorescent microscopy. Representative pictures are shown. (B) GFP fluorescence intensity was measured by FACS. Representative results from 3 independent experiments are shown. (C–E) *Acta2*, *Col1a1*, and *Serpine1* mRNA expression was determined by quantitative real-time PCR. N = 5 in each group. Data are shown as mean ± SD. Significance was determined using 2-way ANOVA with the Bonferroni post hoc analysis. *p < 0.05, **p < 0.005. Representative results from 3 independent experiments are shown. Abbreviations: NTP, Neurotropin; NU, Neurotropin units; OCA, obeticholic acid; pos., positive.

Because TGF-β is a major activator of HSCs and our in vivo mouse data revealed reduced *Tgfb1* gene expression by NTP treatment, we investigated whether NTP inhibits HSC activation in vitro. To take advantage of HSCs isolated from mice expressing green fluorescent protein under the control of the *Col1a1* promoter, we examined the inhibitory effect of NTP on HSC activation using fluorescent microscopy and flow cytometry. Administration of TGF-β increased *Col1a1* reporter activity and α-SMA expression in HSCs, as measured by green fluorescent protein fluorescent signal and α-SMA immunofluorescence intensities (Figures 6A, B). NTP treatment at 0.2 or 0.4 NU/mL 1 hour before TGF-β administration significantly suppressed TGF-β–induced HSC activation (Figures 6A, B). We also performed quantitative real-time PCR analysis of mouse primary HSCs. NTP treatment significantly suppressed mRNA expression of the fibrogenic genes *Acta2* and *Col1a1* (Figures 6C, D). NTP also suppressed the expression of *Serpine1*, a well-known downstream target of TGF-β that encodes plasminogen activator inhibitor 1 (which plays an important role in tissue fibrosis and vascular disease) (Figure 6E). The results of these in vitro studies support our in vivo data, suggesting that NTP suppresses fibrogenic gene expression and liver fibrosis by inhibiting the TGF-β signaling pathway.

### NTP treatment preserves hepatic mitochondrial function in HFD/HFGW-fed mice

**FIGURE 7:**
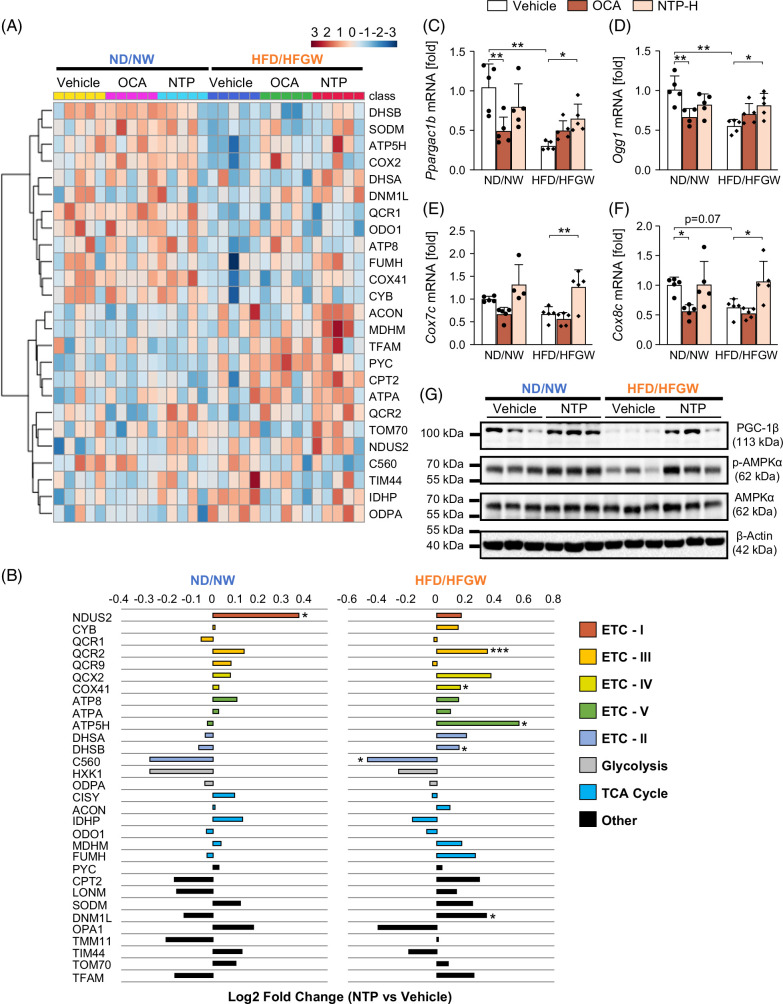
Altered expression of proteins and genes related to mitochondrial function in MASLD/MASH is preserved by NTP treatment. (A) Mitochondrial protein expression was measured using the MitoPlex platform. Protein extracts from liver tissues of our mouse MASLD/MASH model were used. The mice were treated with vehicle, OCA, or NTP. N = 5 per group. The heatmap visualized the 25 top-ranked proteins based on 1-way ANOVA. (B) Comparison of Log2-fold changes in 31 mitochondrial proteins in the liver tissues of mice treated with NTP, compared to vehicle control, as reported by MitoPlex. Significance was determined using an unpaired Student *t* test. *p < 0.05, ***p < 0.001. N = 5 per group. (C–F) Hepatic mRNA expression of *Ppargc1b*, *Ogg1*, *Cox7c*, and *Cox8c* was determined by quantitative real-time PCR. N = 4–5 per group. Data are shown as mean ± SD. Significance was determined using 2-way ANOVA with the Bonferroni post hoc analysis. *p < 0.05, **p < 0.005. (G) Immunoblots for PGC-1β, phospho-AMPKα, total AMPKα, and β-actin using the lysates from liver tissues of our mouse MASLD/MASH model. Three representative protein lysates of each condition were used for immunoblotting. Representative images of 3 independent experiments are shown. Abbreviations: ETC, electron transport chain; HFD/HFGW, high-fat diet with high fructose/glucose drinking water; MASH, metabolic dysfunction–associated steatohepatitis; MASLD, metabolic dysfunction–associated steatotic liver disease; ND/NW, normal chow diet with normal drinking; NTP, Neurotropin; NTP-H, high-dose Neurotropin; OCA, obeticholic acid; TCA, tricarboxylic acid.

We used MitoPlex analysis to evaluate the effects of NTP on hepatic mitochondria. MitoPlex is a high-throughput, reproducible, and quantitative mass spectrometry multiple reaction monitoring-based assay capable of measuring 31 proteins critical to central carbon metabolism and overall mitochondrial function.^24^ Protein data are visualized by a heatmap and comparisons of Log2-fold changes (Figures 7A, B). Among the mitochondrial proteins decreased in HFD/HFGW-fed mice, expression of several proteins was restored by NTP treatment: DHSB, ATP5H, DHSA, COX4-1 (oxidative phosphorylation), SODM (ROS reduction), DNM1L (mitochondrial dynamics), and FUMH (tricarboxylic acid [TCA] cycle) (Figures 7A, B). Expression of these proteins was markedly increased in the NTP treatment group, compared with the vehicle control group, in HFD/HFGW-fed mice: ACON, MDHM (TCA cycle), TFAM (mitochondrial biogenesis), CPT2 (fatty acid transport), ATPA, QCR2, NDUS2 (oxidative phosphorylation), and TOM70 (protein transport) (Figures 7A, B).

As our previous research revealed that NTP treatment increased *Ppargc1b*, *Ogg1*, *Cox7c*, and *Cox8c* expression and prevented palmitic acid (PA)–induced and linoleic acid–induced reduction of these genes in primary mouse hepatocytes,^17^ we examined these genes in liver tissues. NTP treatment of HFD/HFGW-fed mice resulted in increased expression of *Ppargc1b*, *Ogg1*, *Cox7c*, and *Cox8c* in the liver (Figures 7C–F). Subsequently, we validated the protein expression of PGC-1β and phosphorylation of AMPK. HFD/HFGW feeding reduced the protein expression of PGC-1β and phosphorylation of AMPK, whereas NTP treatment increased the protein expression of PGC-1β and restored phosphorylation of AMPK (Figure 7G). Together, our results suggest that NTP prevents HFD/HFGW-induced mitochondrial dysfunction by restoring AMPK phosphorylation and the expression of PGC-1β and mitochondrial proteins related to oxidative phosphorylation, ROS reduction, mitochondrial dynamics and biogenesis, fatty acid transport, and the TCA cycle.

### NTP treatment inhibits FFA-induced lipid accumulation in human hepatocytes

**FIGURE 8:**
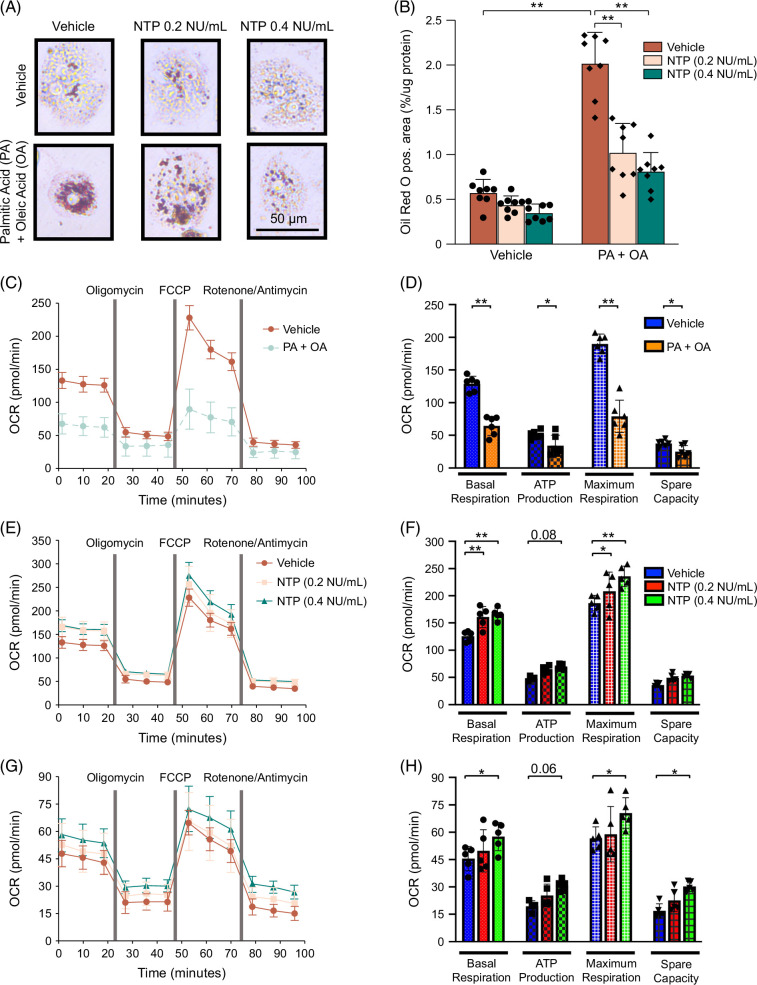
NTP prevents lipid accumulation and improves mitochondrial function in human hepatocytes. Primary human hepatocytes were pretreated with vehicle or NTP (0.2 or 0.4 NU/mL) for 1 hour, followed by treatment with 300 μM palmitic acid (PA) and 300 μM oleic acid (OA) or control (BSA) for an additional 24 hours. Cellular lipid accumulation was examined using Oil Red O staining. (A) Representative microscopic images of Oil Red O staining. (B) Quantification of Oil Red O staining. Data are shown as mean ± SD of 8 high-power fields (×200). Significance was determined using 2-way ANOVA with the Bonferroni post hoc analysis. *p < 0.05, **p < 0.005. Similar results were obtained in 3 independent experiments. Representative results are shown. (C, D) Mitochondrial respiration measured by Seahorse assay in primary human hepatocytes treated with vehicle or 300 μM PA plus 300 μM OA for 25 hours. (C) Kinetics of oxygen consumption rate (OCR) after sequential compound injections. (D) Bar chart highlighting the differences in basal respiration, ATP production, maximal respiration, and spare respiratory capacity between groups. N = 5 per group. Data are shown as mean ± SD. Significance was determined using 2-way ANOVA with the Bonferroni post hoc analysis. *p < 0.05, **p < 0.005. (E, F) Mitochondrial respiration measured by the Seahorse assay in primary human hepatocytes treated with vehicle or NTP (0.2 or 0.4 NU/mL) for 25 hours before measuring OCR. (E) Kinetics of OCR after sequential compound injections. (F) Bar chart highlighting differences in basal respiration, ATP production, maximal respiration, and spare respiratory capacity between groups. N = 5 per group. Data are shown as mean ± SD. Significance was determined using 2-way ANOVA with the Bonferroni post hoc analysis. *p < 0.05, **p < 0.005. (G, H) Mitochondrial respiration measured by Seahorse assay in primary human hepatocytes treated with vehicle or NTP (0.2 or 0.4 NU/mL) for 1 hour, followed by treatment with 300 μM PA and 300 μM OA for an additional 24 hours before determining OCR. (G) Kinetics of OCR after sequential compound injections. (H) Bar chart highlighting differences in basal respiration, ATP production, maximal respiration, and spare respiratory capacity between groups. N = 5 per group. Data are shown as mean ± SD. Significance was determined using 2-way ANOVA with the Bonferroni post hoc analysis. *p < 0.05, **p < 0.005. Representative results from 3 independent experiments are shown. Abbreviations: BSA, bovine serum albumin; FCCP, fluoro-carbonyl cyanide phenylhydrazone; NTP, Neurotropin; pos., positive.

Overall, our findings demonstrated that NTP has great potential to inhibit MASLD development in mice. To translate our mouse findings to humans, we used human primary hepatocytes to evaluate the protective effects of NTP on FFA-induced lipid accumulation in these cells. Because PA and oleic acid (OA) are 2 major FFAs that accumulate in MASH livers, we treated human hepatocytes with PA plus OA for 24 hours. One hour before the challenge with these FFAs, the hepatocytes were treated with 0.2 or 0.4 NU/mL NTP. FFA treatment markedly increased hepatocyte lipid content, as demonstrated by Oil Red O staining, and this increase was significantly inhibited by pretreatment with NTP (Figures 8A, B). Thus, the effects of NTP translate to a human-relevant condition at the cellular level.

We also examined mitochondrial function in human hepatocytes using a Seahorse assay. Compared to vehicle control treatment (bovine serum albumin), PA plus OA treatment led to dramatic deterioration of mitochondrial function, including basal respiration, ATP production, maximum respiration, and spare capacity (Figures 8C, D). In normal human hepatocytes (not exposed to PA and OA), NTP treatment significantly increased basal respiration and maximum respiration but did not affect ATP production or spare capacity (Figures 8E, F). In hepatocytes with PA plus OA exposure, NTP pretreatment increased basal respiration, ATP production, maximum respiration, and spare capacity, compared to the vehicle (Figures 8G, H). Taken together, our data suggest that NTP treatment is beneficial for inhibiting FFA-induced lipid accumulation and improving mitochondrial dysfunction in a human-relevant condition.

## DISCUSSION

Recently, resmetirom, a thyroid hormone receptor β agonist, has been approved by FDA.^5^ The current major management of MASLD still focuses on lifestyle modifications, such as consuming a healthy diet, exercising regularly, and reducing body weight. Liver transplantation is the only option if the disease progresses to cirrhosis. In addition to ongoing clinical trials of new drugs, repurposing existing drugs is another potential option for developing effective MASLD treatment. Repurposing significantly reduces time, effort, and costs because data on drug toxicity, safety, off-target effects, and pharmacokinetics are already available. In the present study, we used a mouse model to investigate the potential of repurposing NTP—an analgesic currently used for pain relief and neuropathic conditions—for the treatment of MASLD. We found that compared with OCA, a widely evaluated farnesoid X-activated receptor agonist with anti-MASLD activity,^23^ oral NTP had similar levels of antisteatotic, anti-inflammatory, hepatoprotective, and antifibrotic effects in our mouse model of MASLD/MASH induced by HFD/HFGW feeding.

Substantial reduction in lipid accumulation and hepatocyte damage can be mediated by the protective effects of NTP against mitochondrial damage and dysfunction. In our previous study, we demonstrated that NTP treatment improved mitochondrial membrane potential and suppressed mitochondrial ROS production.^17^ This effect could be mediated by the NTP promotion of mitochondrial biogenesis and turnover through PGC-1β upregulation and AMPK activation.^17^ In the present study, we used the new proteomics platform MitoPlex to quantify mitochondrial proteins and found that NTP can upregulate the expression of mitochondrial proteins related to oxidative phosphorylation (DHSB, ATP5H, DHSA, COX4, ATPA, QCR2, and NDUS2), the TCA cycle (FUMH, ACON, and MDHM), ROS reduction (SODM), mitochondrial biogenesis (TFAM and DNM1L), and fatty acid transport (CPT2). Upregulation of these proteins could improve mitochondrial function, thereby inhibiting the development of hepatic steatosis and injury induced by HFD/HFGW feeding. This ability to preserve mitochondrial function could be a central mechanism of action of NTP in MASLD.

The anti-inflammatory effects of NTP may also be mediated by its beneficial effects on mitochondria. Moreover, our previous study demonstrated that NTP can inhibit TNFα and IL-1β–induced NF-κB activation,^14^ which is associated with acute-phase inflammatory responses, including IL-6 production. NTP also suppresses TNFα and IL-1β–induced JNK activation and JNK-mediated hepatocyte apoptosis.^14^ In addition, we demonstrated the MASLD-induced phosphorylation of JNK, ERK, and p38 in liver tissues was suppressed by NTP treatment. Thus, the regulation of JNK, ERK, and p38 activation could be additional mechanisms of action for NTP-mediated prevention of MALD/MASH progression.

Suppression of hepatic steatosis, injury, and inflammation could prevent the development of fibrosis secondarily by inhibiting HSC activation. Because NTP exhibited dramatic effects in preventing fibrogenesis, we hypothesized that NTP directly inhibits HSC activation. Indeed, we found that NTP inhibited TGF-β–induced type I collagen and α-SMA expression in HSCs.

Our previous study demonstrated the crucial role of AMPK phosphorylation in NTP-mediated protection against mitochondrial damage.^17^ In the present study, we further demonstrated that in vivo NTP treatment recovered AMPK phosphorylation, which was suppressed in MASLD/MASH livers. One that we can propose the mechanism of action of NTP for its beneficial effect on MASLD/MASH is NTP-induced AMPK activation. NTP contains various bioactive molecules, including nucleic acids, amino acids, and sugars.^10^ However, the biologically active components responsible for its treatment effects have not been determined. NTP also contains molecules, such as adenosine and γ-aminobutyric acid (GABA),^10^ that may have the ability to activate AMPK.^25^^,^^26^ We speculate that the adenosine, GABA, and other components contained in NTP may cooperatively activate AMPK, and this effect could be one of the mechanisms of action of NTP in MASLD. Recent reports showed that activation of GABA receptors can improve lipotoxicity-induced hepatocyte injury in vitro, as well as in a mouse model of MASH fibrosis.^27^^,^^28^ We speculate that adenosine, GABA, and other components contained in NTP may activate their receptor signaling to suppress hepatocyte damage, HSC activation, and fibrosis in MASLD. Further studies are required to determine which receptor signaling can be activated by NTP in liver constituent cells, including hepatocytes, HSCs, and liver immune cells.

Although OCA dramatically inhibits the development of MASLD/MASH, including hepatic steatosis, injury, inflammation, and fibrosis, it has substantial off-target effects, including pruritus and elevated cholesterol levels. The FDA recently rejected OCA as a treatment for precirrhotic fibrosis caused by MASH. In contrast, NTP does not exhibit these off-target effects, which could be a distinct advantage for clinical use.

In summary, the present study clearly demonstrated that NTP has antisteatotic, anti-inflammatory, hepatoprotective, and antifibrotic effects in vivo, similar to those observed with OCA. Mechanisms of action of these effects may include preservation of mitochondrial function and suppression of mitochondrial ROS, NF-κB, and JNK activation. Anti-inflammatory and antifibrotic effects could be a secondary effect of NTP on hepatoprotective and inhibition of mitochondrial dysfunction in hepatocytes. However, because NTP directly inhibited HSC activation and fibrotic response, this direct inhibitory effect on HSC activation could be important. Activation of AMPK and GABA receptors could also play a key role in the improvement of MASLD observed with NTP. Of clinical relevance, patients with chronic pain are frequently candidates for NTP analgesic therapy and also have metabolic syndrome.^29^^–^^32^ While many analgesics, such as NSAIDs, are hepatotoxic, NTP appears to be hepatoprotective instead. Additional clinical studies are required to validate the efficacy of NTP in patients with MASLD. Nonetheless, our results provide new insights regarding the potential repurposing of NTP to manage this important and prevalent condition.

## Supplementary Material
