# Supplementary material for: Repurposing of the analgesic Neurotropin for MASLD/MASH treatment
Source: Hepatol Commun. 2024 Jul 18;8(8):e0480. doi: 10.1097/HC9.0000000000000480 (PMC11262822; doi:10.1097/HC9.0000000000000480)
Supplement: Supplementary file 1 [file hc9-8-e0480-s001.docx]

**Title: Repurposing of the analgesic Neurotropin for MASLD/MASH treatment**

Takashi Tsuchiya, B.S.^1^, So Yeon Kim, Ph.D.1, Michitaka Matsuda, M.D., Ph.D.,^1^, Jieun Kim, Ph.D.1, Alexsandr Stotland, Ph.D.^1^, Mitsuru Naiki, Ph.D.^2^, Ekihiro Seki, M.D., Ph.D.^1,3^

^1^Department of Medicine, Cedars-Sinai Medical Center, Los Angeles, California 90048, USA; ^2^Department of Pharmacological Research, Institute of Bio-Active Science, Nippon Zoki Pharmaceutical Company Ltd., Osaka 541-0046, Japan; ^3^Department of Biomedical Sciences, Cedars-Sinai Medical Center, Los Angeles, California 90048, USA

**SUPPLEMENTARY MATERIALS AND METHODS**

**Reagents**

Hematoxylin and eosin (H-E) staining kit was obtained from Leica Biosystems (Deer Park, IL, USA). Potassium permanganate (Beantown Chemical, Hudson, NH, USA), Sulfuric acid, Sliver nitrate (VWR International, Radnor, PA, USA), Oxalic acid (Aesar (Ward Hill, MA, USA), Ferric ammonium sulfate, 10% PBS-buffered formalin, Sodium hydroxide (VWR), Aluminum sulfate (Beantown Chemical), Sodium thiosulfate (VWR), and Nuclear fast red for reticulin staining (TCI America, Portland, OR, USA) were used. Oil Red O staining solution was obtained from Thermo Scientific (Thermo Scientific, MA, USA). The antibody for F4/80 was purchased from BioLegend (San Diego, CA, USA). The antibody for alpha smooth muscle actin (αSMA) was obtained from Sigma-Aldrich (St. Louis, MO, USA). The kits and reagents for Seahorse assay were from Agilent technology (Santa Clara, CA, USA).

**Histology**

All tissues were fixed in 10% PBS-buffered formalin and embedded in paraffin. The slides were deparaffinized with xylene and dehydrated in gradient ethanol. H-E, and Reticulin staining were performed. Reticulin stains were performed using modified Gordon & Sweet's staining protocol(1). Solutions were freshly prepared before use. Stained reticulin fibers were black against a red-pink (Nuclear Fast Red counterstain) background. H-E and Reticulin positive area was measured on 10 high power (x100) fields/slide. Images were captured on a DMi8 inverted microscope (Leica) and analyzed by ImageJ software.

**Measurement of serum and hepatic TG, Cholesterol, and FFA contents**

Serum and hepatic TG, cholesterol, and FFA concentrations were determined using TG GPO liquid reagent sets (Pointe Scientific Inc.), Wako total cholesterol E reagents (FUJIFILM Medical Systems U.S.A. Inc.), and a FFA assay kit (Abcam, Cambridge, UK) according to the manufacturer’s protocol, respectively. As for liver TG, cholesterol, and FFA contents were normalized to the total protein concentration and expressed as milligrams per grams protein, milligrams per grams protein, nanomoles per grams protein, respectively.

**Measurement of serum ALT, AST, and insulin concentrations**

Kits for measuring serum ALT and AST levels were measured at 340 nanometers using CLARIO star (BMG LABTECT, Ortenberg, Germany), and assessed by Kinetic method. Insulin resistance was assessed by HOMA-IR [fasting glucose (mmol/l) × fasting insulin (µU/ml) ÷ 22.5] by measuring serum insulin (Crystal Chem) and blood glucose levels.

**Human hepatocytes culture and treatment**

Primary human hepatocytes (Lot # HU8307) were purchased from Gibco (Gibco, Frederick, MD). Vials were thawed at a 37 °C water bath, and the contents were quickly transferred to the 50 mL tube containing prewarmed 3 mL of the Hepatocytes Medium (William's E Medium without phenol red with 1 % Pen/Strep, 1 % L-GlutaMAX, 1 % ITS+premix, 0.05 mg/mL of ascorbic acid, 1 μM dexamethasone, 5 % FBS) and rinsed with 1 mL of the Medium. The tube was gently agitated by swirling, and the suspension was brought up to 35 mL with the Hepatocyte Medium. Finally, 15 mL of 90 % Percoll solution (90 % Percoll solution and 10 % 10X DPBS) was added to bring up the total volume to 50 mL. The tube was gently inverted 3 times to mix the cell suspension, followed by centrifugation at 96 g for 6 minutes at room temperature. The resultant supernatant was carefully aspirated, and the undisturbed cell pellet was resuspended with 3 to 5 mL of the medium by rotating the tube gently. The suspension was brought up to 50 mL. with the fresh medium, followed by centrifugation at 72 g for 4 minutes at room temperature. The supernatant was aspirated down to 1 to 2 mL. Trypan blue was used to count and measure the total cell yield and viability (cells with >90% viability were used for the experiments) under a microscope. The cells were seeded at a cell density of 80 % density to the type I collagen coated culture plates. Four hours after seeding the primary human hepatocytes, the medium was changed to serum-free Hepatocyte Medium. After an overnight incubation, the cells were then treated with NTP (0.2 or 0.4 NU/ml), for 1 hour, followed by vehicle (non-free fatty acid BSA) or 300 μM PA plus 300 μM OA for an additional 24 hours(2). After these treatments, cells were used for Oil Red O staining and Seahorse analysis.

**Oil red O staining**

For animal studies, frozen liver sections were air-dried at room temperature for 60 minutes and fixed in 10% PBS-buffered formalin for 10 minutes. The fixed sections were rinsed with 60% isopropanol and stained with a freshly prepared Oil Red O working solution for 15 minutes. After washing with distilled water, the slides were mounted with aqueous mounting medium (Vector Laboratories, Newar, CA, USA). Oil red O positive area was measured on 10 high power (x200) fields/slide. Images were captured on a DMi8 inverted microscope (Leica) and analyzed by ImageJ software.

For in vitro studies, after a 24 hour-treatment with NTP, human primary hepatocytes (Gibco) were washed twice with PBS, fixed with 10% PBS-buffered formalin for 1 hour, and then stained with a freshly prepared Oil red O working solution for 10 minutes at room temperature, followed by washing in distilled water three times. Cell images were captured with a Leica DMi8. Oil red O positive area was measured on 8 high power (x200) fields/slide. Images were captured on a DMi8 inverted microscope (Leica) and analyzed by ImageJ software. After image capture, isopropanol was added to each well, and the extracted dye was measured at 500 nm for quantification.

**RNA extraction and qPCR analysis**

Total RNA was isolated from cells or tissues with TRIzol (Thermo Fisher Scientific, Cat# 15596018) in accordance with the manufacturer’s instructions. The total RNA extracted from tissue samples using TRIzol was purified with NucleoSpin RNA kit (MACHEREY-NAGEL, Cat# 740955.50) according to the company’s protocol. Reverse transcription reactions were performed using iScript Reverse Transcription Supermix (Bio-Rad Laboratories, Cat# 1708841). Then, qRT-PCR was conducted in 96-well plates using iTaq Universal SYBR Green Supermix (Bio-Rad Laboratories, Cat# 1725122) on a CFX96 real-time system (Bio-Rad Laboratories). *18s* was used as a housekeeping gene for detection of mRNA expression. The comparative CT method and standard curve method were used to calculate the fold change and relative abundance of gene expression.

The mouse-specific primers for qPCR were as follows: *Scd-1, Dgat-1, Dgat-2, Srebp1c, Cxcl1, Ccl2, Cxcl5, Tnf, Il6, Col1a1, Col3a1, Col4a1, Timp1, Tgf-b, Acta2, Serpine1, Cox7x, Cox8c, Ogg1, Ppargc1b, 18s*. See Supplemental Table S2.

**Flow cytometry**

Primary HSCs were cultured in DMEM containing 10% FBS overnight. The next day, the medium was changed to serum-free DMEM, after which the cells were incubated overnight. The cells were then treated with NTP (0.2 or 0.4 NU/ml) for 1 hour, followed by 5 ng/mL TGF-β for an additional 24 hours. The treated cells were suspended in 1× PBS with 3% FBS staining buffer and analyzed by flow cytometry (BD LSR II Cell Analyzer, BD Biosciences, San Jose, CA, USA).

**Immunoblotting**

Protein extracts from liver tissues were electrophoresed and then blotted. Blots were incubated with antibodies for phospho-JNK, JNK, phospho-ERK, ERK, phospho-p38, p38, phospho-AMPKα, and AMPKα (Cell Signaling Technologies), PGC-1β (Abcam), and β-actin (Sigma-Aldrich, St. Louis, MO).

**MitoPlex protein assay**

A tier 2 level targeted proteomic analysis was described previously (3) and was used to quantify mitochondrial proteins between ND/NW-fed mouse liver and HFD/HFGW-fed mouse liver samples. Tissues were lysed in 8 M urea dissolved in 1 M ammonium bicarbonate buffer, pH 8.0. Following extraction, protein concentration was assayed using a Pierce BCA assay kit (Thermo Fisher Scientific). Thirty-five micrograms of protein from each sample were aliquoted for digestion, and each sample was reduced using 10 mM DTT and subsequently alkylated with 100 mM iodoacetamide. Samples were diluted with 200 mM ammonium bicarbonate buffer and supplemented with 10 % acetonitrile, to a final urea concentration of 2 M. Trypsin was added at a ratio of 1 μg to 35 μg of total protein and samples were left to incubate overnight at 37 ºC. Digestion was quenched with 1 % trifluoroacetic acid (TFA), and samples were desalted on Nest C18 tips (NestGroup, Southborough, MA). Peptides were dried to completion and resuspended in a solution of 0.1 % formic acid in H_2_O into which a 1:250 dilution of stable isotope-labeled reference peptides derived for targeted mitochondrial protein analysis as described in detail (3) were added. A total of 8 μg of digested peptides, injected twice as duplicate technical replicates, were separated on a Prominence UFLCXR HPLC system (Shimadzu Corp., Kyoto, Japan) with a Waters Xbridge BEH30 C18 2.1 mm x 100 mm, 3.5-μm column (Waters, Framingham, MA) flowing at 0.25 ml/min and 36 °C coupled to a QTRAP 6500 (SCIEX, Framingham, MA). Mobile phase A consisted of 2 % acetonitrile, 98 % water, and 0.1 % formic acid, and mobile phase B consisted of 95 % acetonitrile, 5 % water, and 0.1 % formic acid. After loading, the column was equilibrated with 5 % B for 5 minutes. Peptides were then eluted over 30 minutes with a linear 5-35% gradient of buffer B. The column was washed with 98 % B for 10 minutes and then returned to 5 % B for 5 minutes before loading the next sample. A scheduled, targeted acquisition method optimized for each peptide was used to monitor fragments within a 2-minutes window of the optimized retention time. Raw data were processed using the Skyline software package (Skyline Daily, version 21.1.0.146) to select peak boundaries and quantify the area under the curve for each fragment monitored. Peptides were manually inspected in Skyline for quality and excluded from further analysis if no discernable peak was observed. Skyline automated peak integration was used to define peak boundaries. The full Skyline data file has been shared on the PRIDE Proteomics web server, including the data from acquired but excluded peptide peak groups ND/NW OCA and HFD/HFGW OCA for Figure 6F only. After processing in Skyline, fragment level data were exported as comma-separated values and further processed using a custom R script (3). Only fragments with 20 % coefficient of variation (CV) across the heavy standard peptides were used for quantification. The abundance ratios of endogenous to heavy standard for all quantified fragments for each peptide from each protein were averaged, and peptide level abundance ratios were further averaged to yield a final abundance ratio for each of the proteins monitored. The technical replicate abundance ratio was averaged for each sample to yield the final protein level measurements. Results were analyzed in MetaboAnalyst 5.0 (<http://www.metaboanalyst.ca>).

**Methods for Seahorse Bioanalyzer**

Mitochondrial respiration was examined by measuring the oxygen consumption rate (OCR) using the Seahorse XF24 extracellular flux analyzer (Seahorse Bioscience, Billerica, MA, USA) (4). Primary human hepatocytes were seeded and treated as described above in XF24 cell culture plates (Seahorse Bioscience) coated with type I collagen. One day before the assay, all wells of the sensor plate were thoroughly hydrated with 500 mL of XF Calibrant buffer (Seahorse Bioscience), and incubated in a non-CO_2_ incubator at 37 ºC for overnight. Before starting the assay, the medium was replaced with bicarbonate-free low-buffered medium (Seahorse Bioscience) containing 25 mM glucose, 2 mM glutamine, 1 mM sodium pyruvate (pH 7.4). After calibration, the plates were placed into the XF24 analyzer, and the OCR was evaluated by sequential injection of 5 μM oligomycin, 8 μM carbonyl cyanide-4-(trifluoromethoxy) phenylhydrazone (FCCP), and 0.5 μM rotenone/antimycin A. OCR was automatically calculated by the analyzer.

**References**

1. Karapinar M, Gonul SA. Survival of Yersinia enterocolitica and Escherichia coli in spring water. Int J Food Microbiol 1991;13:315-319.

2. Jang KJ, Otieno MA, Ronxhi J, Lim HK, Ewart L, Kodella KR, Petropolis DB, et al. Reproducing human and cross-species drug toxicities using a Liver-Chip. Sci Transl Med 2019;11.

3. Stotland AB, Spivia W, Orosco A, Andres AM, Gottlieb RA, Van Eyk JE, Parker SJ. MitoPlex: A targeted multiple reaction monitoring assay for quantification of a curated set of mitochondrial proteins. J Mol Cell Cardiol 2020;142:1-13.

4. Lyra-Leite DM, Andres AM, Petersen AP, Ariyasinghe NR, Cho N, Lee JA, Gottlieb RA, et al. Mitochondrial function in engineered cardiac tissues is regulated by extracellular matrix elasticity and tissue alignment. Am J Physiol Heart Circ Physiol 2017;313:H757-H767.

**Supplemental Table S1 Body/liver/Liver body ratio/Blood glucose/ALT/AST and lipid level at 12 wk after the ND/NW or HFD/HFGW diet feeding**

|  | ND/NW | | | | HFD/HFGW | | | | |
| --- | --- | --- | --- | --- | --- | --- | --- | --- | --- |
|  | Vehicle | OCA | NTP-L | NTP-H | | Vehicle | OCA | NTP-L | NTP-H |
|  | **WT mice**  **(*n* = 10)** | **WT mice**  **(*n* = 10)** | **WT mice**  **(*n* = 10)** | **WT mice**  **(*n* = 9)** | | **WT mice**  **(*n* = 10)** | **WT mice**  **(*n* = 10)** | **WT mice**  **(*n* =10)** | **WT mice**  **(*n* = 10)** |
| Body weight, g,  start, wk = 0 | 26.64 ± 1.71 | 26.3 ± 1.72 | 26.94 ± 1.51 | 26.75 ± 1.53 | | 27.64 ± 1.56 | 27.91 ± 2.99 | 26.48 ± 1.69 | 27.01 ± 2.04 |
| Body weight, g,  strat treatment, wk = 6 | 30.10 ± 2.46 | 30.63 ± 3.03 | 31.5 ± 2.12 | 31.25 ± 2.38 | | 44.51 ± 5.23* | 43.71 ± 3.9* | 43.10 ± 4.81* | 44.03 ± 3.74* |
| Body weight, g,  end, wk = 12 | 31.35 ± 2.56 | 30.88 ± 2.9 | 33.39 ± 2.21 | 33.1 ± 2.76 | | 48.15 ± 4.28* | 48.57 ± 4.53* | 47.75 ± 2.91* | 48.86 ± 2.46* |
| Liver weight, g | 1.16 ± 0.08 | 1.07 ± 0.09 | 1.24 ± 0.1 | 1.14 ± 0.12 | | 2.36 ± 0.42* | 1.88 ± 0.37* | 1.89 ± 0.49* | 1.97 ± 0.19* |
| Liver/Body weight, % | 0.04 ± 0.003 | 0.03 ± 0.002 | 0.04 ± 0.002 | 0.04 ± 0.001 | | 0.04 ± 0.005 | 0.04 ± 0.005 | 0.04 ± 0.008 | 0.04 ± 0.003 |
| Blood glucose, mg/dL | 118.56 ± 13.84 | 106.2 ± 17.43 | 112.4 ± 22.43 | 115.22 ± 19.44 | | 128.53 ± 25.33 | 136.64 ± 42.34 | 96.3 ± 15.23† | 136.75 ± 25.22 |
| ALT, U/L | 14.47 ± 3.01 | 12.03 ± 2.44 | 8.56 ± 1.15 | 9.78 ± 1.84 | | 91.56 ± 14.12* | 50.51 ± 12.82*† | 60.66 ± 22.76*† | 51.23 ± 9.76*† |
| AST, U/L | 23.22 ± 3.88 | 24.78 ± 6.32 | 17.55 ± 2.57 | 24.66 ± 2.31 | | 78.46 ± 8.05* | 48.51 ± 8.68*† | 57.23 ± 13.60*† | 55.91 ± 5.70*† |
| **Serum** |  |  |  |  | |  |  |  |  |
| Triglyceride, mg/dl | 43.82 ± 9.63 | 36.86 ± 7.63 | 56.14 ± 8.67 | 47.57 ± 15.43 | | 69.22 ± 8.61* | 61.91 ± 18.36* | 84.50 ± 10.48* | 58.80 ± 6.06* |
| Total cholesterol, mg/dl | 83.19 ± 7.26 | 61.79 ± 13.92 | 83.08 ± 9.45 | 79.39 ± 9.61 | | 287.26 ± 24* | 150.27 ± 30.47*† | 222.46 ± 51.76*† | 257.71 ± 33.16* |
| Free fatty acid, nmol/ul | 1.32 ± 0.39 | 1.33 ± 0.32 | 0.93 ± 0.22 | 1.23 ± 0.22 | | 2.57 ± 0.3* | 2.23 ± 0.51* | 1.31 ± 0.27† | 2.18 ± 0.28* |
| **Liver** |  |  |  |  | |  |  |  |  |
| Triglyceride, mg/g | 41.07 ± 26.5 | 38.4 ± 7.43 | 34.65 ± 16.17 | 41.01 ± 10.48 | | 259.08 ± 73.71* | 156.45 ± 35.83*† | 181.66 ± 46.37*† | 167.05 ± 37.51*† |
| Total cholesterol, mg/g | 4.92 ± 1.42 | 6.29 ± 1.08 | 5.02 ± 1.51 | 5.21 ± 0.43 | | 10.65 ± 4.78* | 16.95 ± 6.21*† | 13.98 ± 4.23* | 11.29 ± 4.63* |
| Free fatty acid, nmol/g | 0.96 ± 0.26 | 1.13 ± 0.31 | 1.08 ± 0.25 | 1.25 ± 0.15 | | 11.81 ± 4.94* | 5.95 ± 1.65*† | 7.31 ± 2.4*† | 6.99 ± 2.64*† |
| HOMA-IR | 9.36 ± 5.76 | 6.17 ± 2.84 | 7.24 ± 5.05 | 6.98 ± 4.68 | | 26.68 ± 9.39* | 28.32 ± 16.06* | 22.50 ± 11.82* | 26.80 ± 10.02* |

Values are means ± SD.

*Significantly different from wild-type (WT)-normal diet + normal water-defined (ND/NW), P < 0.05.

†Significantly different from WT-high fat diet + high fructose and glucose water-defined (HFD/HFGW), P < 0.05.

HOMA-IR, homeostasis model assessment of insulin resistance.

| Gene name | Forward | Reverse |
| --- | --- | --- |
| *Scd* | 5’ GCTCTACACCTGCCTCTTCG 3’ | 5’ CAGCCGAGCCTTGTAAGTTC 3’ |
| *Srebf1* | 5’ GAACAGACACTGGCCGAGAT 3’ | 5’ GAGGCCAGAGAAGCAGAAGAG 3’ |
| *Dgat1* | 5’ GACGGCTACTGGGATCTGA 3’ | 5’ TCACCACACACCAATTCAGG 3’ |
| *Dgat2* | 5’ GAAGATGTCTTGGAGGGCTG 3’ | 5’ GGCAGCGAAAACAAGAATAA 3’ |
| *Cxcl1* | 5’ GCTTGAAGGTGTTGCCCTCAG 3’ | 5’ AAGCCTCGCGACCATTCTTG 3’ |
| *Ccl2* | 5’ ATTGGGATCATCTTGCTGGT 3’ | 5’ CCTGCTGTTCACAGTTGCC 3’ |
| *Cxcl5* | 5’ TGATCCCTGCAGGTCCACA 3’ | 5’ CTGCGAGTGCATTCCGCTTA 3’ |
| *Tnf* | 5’ AGGGTCTGGGCCATAGAACT 3’ | 5’ CCACCACGCTCTTCTGTCTAC 3’ |
| *Il6* | 5’ ACCAGAGGAAATTTTCAATAGGC 3’ | 5’ TGATGCACTTGCAGAAAACA 3’ |
| *Col1a1* | 5’ TAGGCCATTGTGTATGCAGC 3’ | 5’ ACATGTTCAGCTTTGTGGACC 3’ |
| *Col3a1* | 5’ TAGGACTGACCAAGGTGGCT 3’ | 5’ GGAACCTGGTTTCTTCTCACC 3’ |
| *Col4a1* | 5’ CACATTTTCCACAGCCAGAG 3’ | 5’ GTCTGGCTTCTGCTGCTCTT 3’ |
| *Timp1* | 5’ AGAGTGTCTGCGGATACTTCC 3’ | 5’ CCAACAGTGTAGGTCTTGGTG 3’ |
| *Tgfb1* | 5’ GTGGAAATCAACGGGATCAG 3’ | 5’ ACTTCCAACCCAGGTCCTTC 3’ |
| *Acta2* | 5’ GTGTTGCCCCTGAAGAGCAT 3’ | 5’ GCTGGGACATTGAAAGTCTCA 3’ |
| *Serpine1* | 5’ CCTCTTCCACAAGTCTGATGGC 3’ | 5’ GCAGTTCAACAACGTCATACTCG 3’ |
| *Ppargac1b* | 5’ TCCTGTAAAAGCCCGCAGTAT 3’ | 5’ GCTCTGGTAGGGGCAGTGA 3’ |
| *Ogg1* | 5’ CTGCCTAGCAGCATGAGACAT 3’ | 5’ CAGTGTCCATACTTGATCTGCC 3’ |
| *Cox7c* | 5’ ATGTTGGGCCAGAGTATCCG 3’ | 5’ GATTGCAGAAGAGGTGACTGG 3’ |
| *Cox8c* | 5’ TTCCTGCTTCGTGTGTTGTC 3’ | 5’ GCTCTGGTAGGGGCAGTGA 3’ |
| *18S* | 5’ AGTCCCTGCCCTTTGTACACA 3’ | 5’ CGATCCGAGGGCCTCACTA 3’ |

**Supplemental Table S2. Mouse quantitative real-time PCR primer information**

*All primers purchased from Integrated DNA Technologies
